# Transactions of the New York Academy of Medicine

**Published:** 1884-12

**Authors:** 


*Transactions of the New York Academy of Medicine.* Second Series. Vol. I., 1874. Vol. II., 1876. Vol. III., 1883. New York: APPLETON & Co.

These three octavo volumes, printed and bound in the handsome manner which we are so pleasantly familiar with in American publications, contain papers read before the New York Academy of Medicine during the past ten years. Why two years should elapse between the first and second volumes, and seven between the second and third, we cannot say; but we will say that if there were many papers read and not printed which came near the standard of those before us, we should have gladly seen those published also. All the papers in the *Transactions* are worthy of study; some are of a high order of excellence, a few especially so. Among these last we would instance some remarkable papers on "Spectrum Analysis as applied to Medicine," in the first and second volumes. The last volume is the least excellent of the three; we trust that this is no indication of falling off in the work of the Academy.

